# Applied deep learning in neurosurgery: identifying cerebrospinal fluid (CSF) shunt systems in hydrocephalus patients

**DOI:** 10.1007/s00701-024-05940-3

**Published:** 2024-02-07

**Authors:** Thomas Rhomberg, Felipe Trivik-Barrientos, Arsany Hakim, Andreas Raabe, Michael Murek

**Affiliations:** 1 Department of Neurosurgery, Inselspital, University Hospital Bern, Bern, Switzerland; 2 Department of Neurosurgery and Neurorestoration, Klinikum Klagenfurt Am Wörthersee, Klagenfurt, Austria; 3 Department of Neurosurgery, Landesklinikum Wiener Neustadt, Wiener Neustadt, Austria; 4 Department of Neuroradiology, Inselspital, University Hospital Bern, Bern, Switzerland

**Keywords:** Deep learning, AI, Transfer learning, Hydrocephalus, Cerebrospinal fluid shunt, CSF shunt, Ventriculoperitoneal shunt, X-ray

## Abstract

**Background:**

Over the recent decades, the number of different manufacturers and models of cerebrospinal fluid shunt valves constantly increased. Proper identification of shunt valves on X-ray images is crucial to neurosurgeons and radiologists to derive further details of a specific shunt valve, such as opening pressure settings and MR scanning conditions. The main aim of this study is to evaluate the feasibility of an AI-assisted shunt valve detection system.

**Methods:**

The dataset used contains 2070 anonymized images of ten different, commonly used shunt valve types. All images were acquired from skull X-rays or scout CT-images. The images were randomly split into a 80% training and 20% validation set. An implementation in Python with the FastAi library was used to train a convolutional neural network (CNN) using a transfer learning method on a pre-trained model.

**Results:**

Overall, our model achieved an F1-score of 99% to predict the correct shunt valve model. F1-scores for individual shunt valves ranged from 92% for the Sophysa Sophy Mini SM8 to 100% for several other models.

**Conclusion:**

This technology has the potential to automatically detect different shunt valve models in a fast and precise way and may facilitate the identification of an unknown shunt valve on X-ray or CT scout images. The deep learning model we developed could be integrated into PACS systems or standalone mobile applications to enhance clinical workflows.

**Supplementary Information:**

The online version contains supplementary material available at 10.1007/s00701-024-05940-3.

## Introduction

Placement of cerebrospinal fluid (CSF) shunt systems to treat hydrocephalus is a common neurosurgical procedure and a life-saving treatment for many patients. In the USA, the incidence of CSF shunt procedures is approximately 7 per 100,000 individuals [1]. In Europe, the incidence varies, with rates of 3.11 per 100,000 in Ireland and the UK [4], and up to 6.942 per 100,000 inhabitants annually in Germany [14]. These implants drain excess fluid from the brain to another part of the body to relieve intracranial pressure. Current CSF shunts contain three main components: a brain catheter for inflow, a valve that regulates the passage of CSF, and an outflow catheter that drains CSF into the abdomen or the heart. Radiopaque markers give each shunt valve model a specific appearance on radiographs, revealing essential details such as pressure settings or susceptibility to magnetic field interference [3, 14, 15].

Therefore, correctly identifying the type of CSF shunt system is essential in clinical practice as it can directly impact patient safety. Insufficient image quality due to motion artifacts or insufficient image resolution as well as adjacent radiopaque structures can render classifying a CSF shunt system a tedious and challenging task. Furthermore, there has been an increase in the number of manufacturers and models of CSF shunt systems in recent years, and new models are constantly being introduced [3]. Advances in computer vision with deep learning models have greatly improved image recognition capabilities. Deep learning is a subfield of artificial intelligence that employs neural networks with multiple layers to analyze various forms of data. These networks are designed to automatically and adaptively learn from data patterns, thereby enabling increasingly accurate interpretations of new data. In the medical imaging context, deep learning models can autonomously analyze images to identify particular structures or detect pathological abnormalities in some cases even surpassing humans in speed and accuracy [7, 8, 13, 18]. Deep learning models have recently been used successfully in a wide range of medical imaging tasks, including tissue segmentation and lesion detection [19]. This has shown promising potential for aiding diagnostic and treatment planning procedures as well as improving surgical safety [16]. Using such technology for automatic identification of CSF shunt systems could help clinicians with this tedious task in routine clinical practice.

We propose a mobile, low-cost approach that uses deep learning to accurately identify CSF shunt systems on plain radiographs. Our model was trained on 2070 images of ten commonly used CSF shunt valve models from two European neurosurgical centers.

## Material and methods

The manuscript was prepared according to the checklist for evaluation of radiomics (CLEAR) and the checklist for artificial intelligence in medical imaging (CLAIM) guidelines [12, 17].

In our approach, the image classification task was accomplished exclusively through feature extraction using a convolutional neural network (CNN). This method capitalizes on the CNN’s ability to identify and learn relevant features from the entire image for accurate classification of shunt valves. We did not employ additional techniques such as image segmentation to isolate specific regions or objects within the images, as our model’s performance was predicated on analyzing the complete image context.

**Fig. 1:**
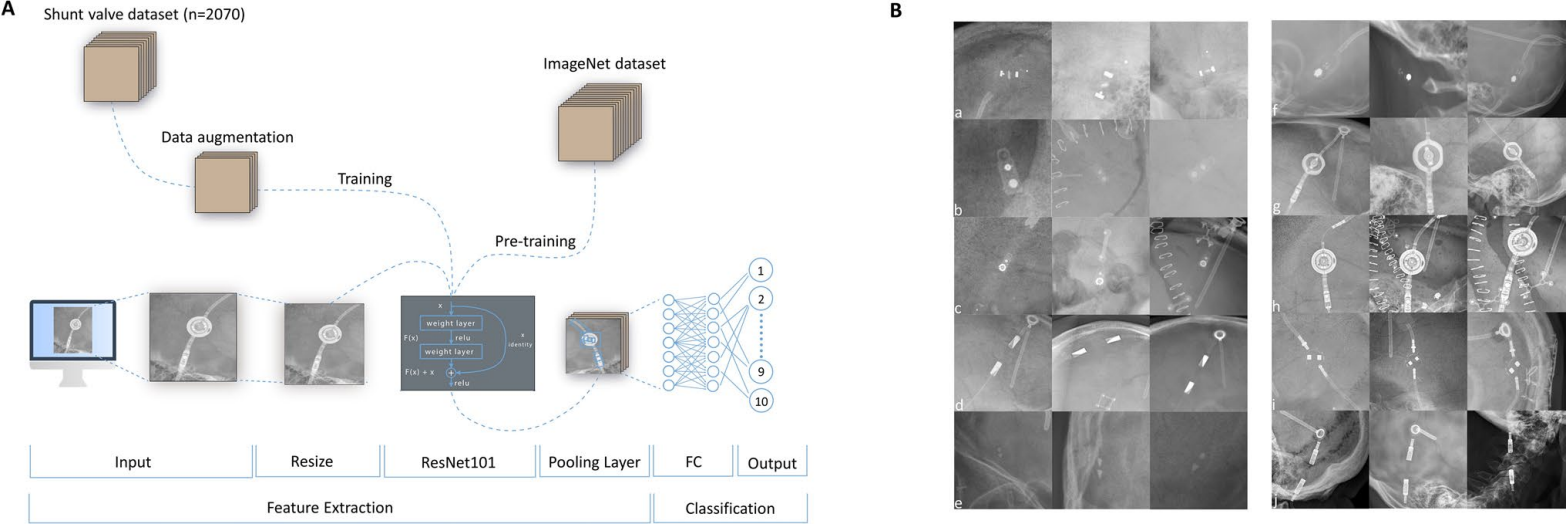
**A** Schematic overview of the neural network model and its components (FC, fully connected layer). **B** Sample images of our dataset with three representative images for each shunt valve: (a) Codman Certas Plus, (b) Codman Hakim Precision Fixed Pressure, (c) Codman Hakim Programmable, (d) Integra DP, (e) Medtronic PS Medical Delta, (f) Medtronic PS Medical Strata, (g) Miethke proGAV 1, (h) Miethke proGAV 2, (i) Sophysa Sophy Mini SM8, and (j) Codman Certas Plus

To accomplish this, a CNN with transfer learning technique from a pre-trained model on ImageNet [2] data was trained using 80% of the image data (Fig. 1). On the remaining 20% of the images, the neural network’s performance was measured to determine accuracy, recall, and precision. In addition, we calculated the F1-score to account for the class imbalance in our dataset. The dataset consisted of 2070 anonymized images of ten different commonly used shunt valve types, collected from two neurosurgical centers in Switzerland and Austria (Table 1). Notably, this dataset had not been used previously for training any AI models. The sole inclusion criterion was the presence of a CSF shunt valve that was visible and identifiable by the authors on an X-ray or CT scout image. The images were anonymized by removing metadata containing patient information and cropping them to prevent any visible patient data from being included (Fig. 1). All images were acquired from skull X-rays or CT scout images, and confounding factors such as bony structures, catheters, craniotomy plates, and skin staples were included. The screenshots were acquired with SnagIt (version 2021.4.3) and saved as.png files. The.png files had an 8-bit depth with alpha RGB channels and no interlacing.

**Table 1:** Dataset composition

Shunt valve	Training set (*n*)	Validation set (*n*)	Total images
Codman Certas Plus	129	26	155
Codman Hakim Precision Fixed Pressure	49	9	58
Codman Hakim Programmable	383	95	478
Integra DP	65	15	80
Medtronic PS Medical Delta	43	17	60
Medtronic PS Medical Strata	231	73	304
Miethke proGAV 1	323	76	399
Miethke proGAV 2	352	86	438
Sophysa Sophy Mini SM8	26	7	33
Integra Spitz-Holter	56	9	65
Combined dataset	1657	413	2070

An implementation in Python with the FastAi library [10] was used to resize all 2070 CSF shunt valve images to 460 × 460 pixels.

Four different data augmentation methods were used to enhance the training set by artificially creating new training data from existing training data. Images were resized to 224 × 224 pixels by a squishing algorithm. The resulting images were flipped vertically or horizontally and randomly rotated up to 355°. The contrast of the images was adjusted by up to 5% with a probability of 75%.

The neural network model was built using Python with the FastAi framework version 2.5.3 based on PyTorch [10]. We used a pre-trained convolutional neural network (ResNet-101[9]) 101 layers deep, consisting of stacked ResNet building blocks, a pooling layer, and a fully connected layer. The pre-trained weights of the layers were from the PyTorch library, which was trained on data from ImageNet. The first layer had an image input size of 224 × 224, and the combined neural network consisted of 44,611,648 trainable parameters. The model was trained with Google Colab using an NVIDIA Tesla T4 GPU.

The dataset was uniformly and randomly split in accordance with the shunt valve model’s distribution, allocating 80% of the images for the training set and 20% for validation. Training images were set up with a batch size of 48. A flattened cross-entropy function was used as a loss function. The pre-trained backbone was frozen for the first training epoch, and a base learning rate of 0.002 was used. The pre-trained backbone was unfrozen for the subsequent training epochs, and the learning rate was optimized with an Adam optimizer [11]. In total, the model was trained for 65 epochs.

## Results

**Fig. 2:**
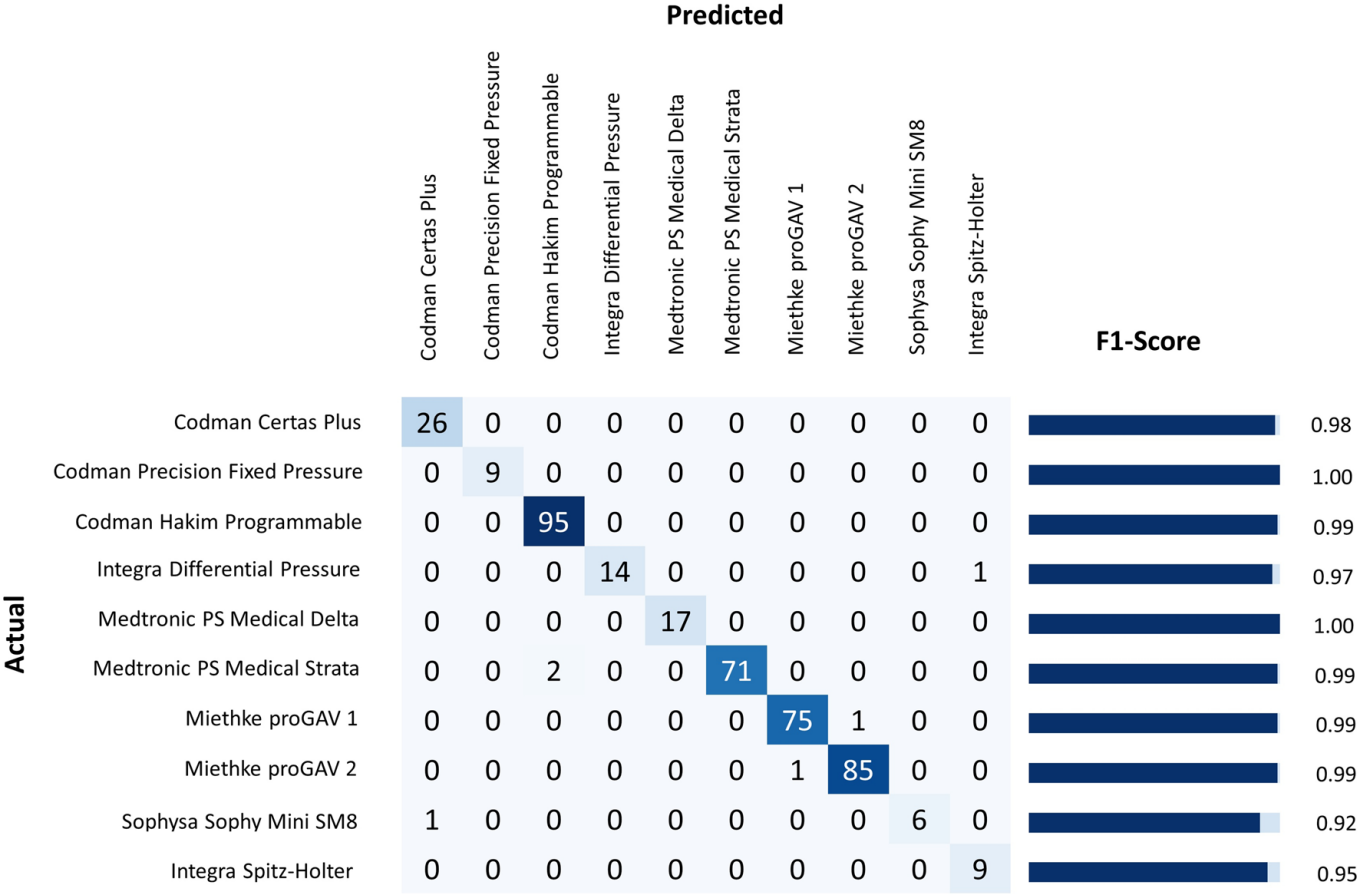
Confusion matrix for the actual and predicted shunt valve models in the validation dataset with its corresponding F1-score

On 413 of the validation images, our model achieved an overall accuracy of 99% with a weighted average F1-score of 99%. Breaking down the performance metric for each of the ten CSF shunt valves (Fig. 2 and Table 2), we achieved the following F1-scores: Codman Certas Plus (98%), Codman Hakim Precision Fixed Pressure (100%), Codman Hakim Programmable (99%), Integra DP (97%), Medtronic PS Medical Delta (100%), Medtronic PS Medical Strata (99%), Miethke proGAV 1 (99%), Miethke proGAV 2 (99%), Sophysa Sophy Mini SM8 (92%), and Integra Spitz-Holter (95%).

**Table 2:** Model performance metrics

Shunt valve	Precision	Recall	F1-score
Codman Certas Plus	0.96	1.00	0.98
Codman Hakim Precision Fixed Pressure	1.00	1.00	1.00
Codman Hakim Programmable	0.98	1.00	0.99
Integra DP	1.00	0.93	0.97
Medtronic PS Medical Delta	1.00	1.00	1.00
Medtronic PS Medical Strata	1.00	0.97	0.99
Miethke proGAV 1	0.99	0.99	0.99
Miethke proGAV 2	0.99	0.99	0.99
Sophysa Sophy Mini SM8	1.00	0.86	0.92
Integra Spitz-Holter	0.90	1.00	0.95

**Fig. 3:**
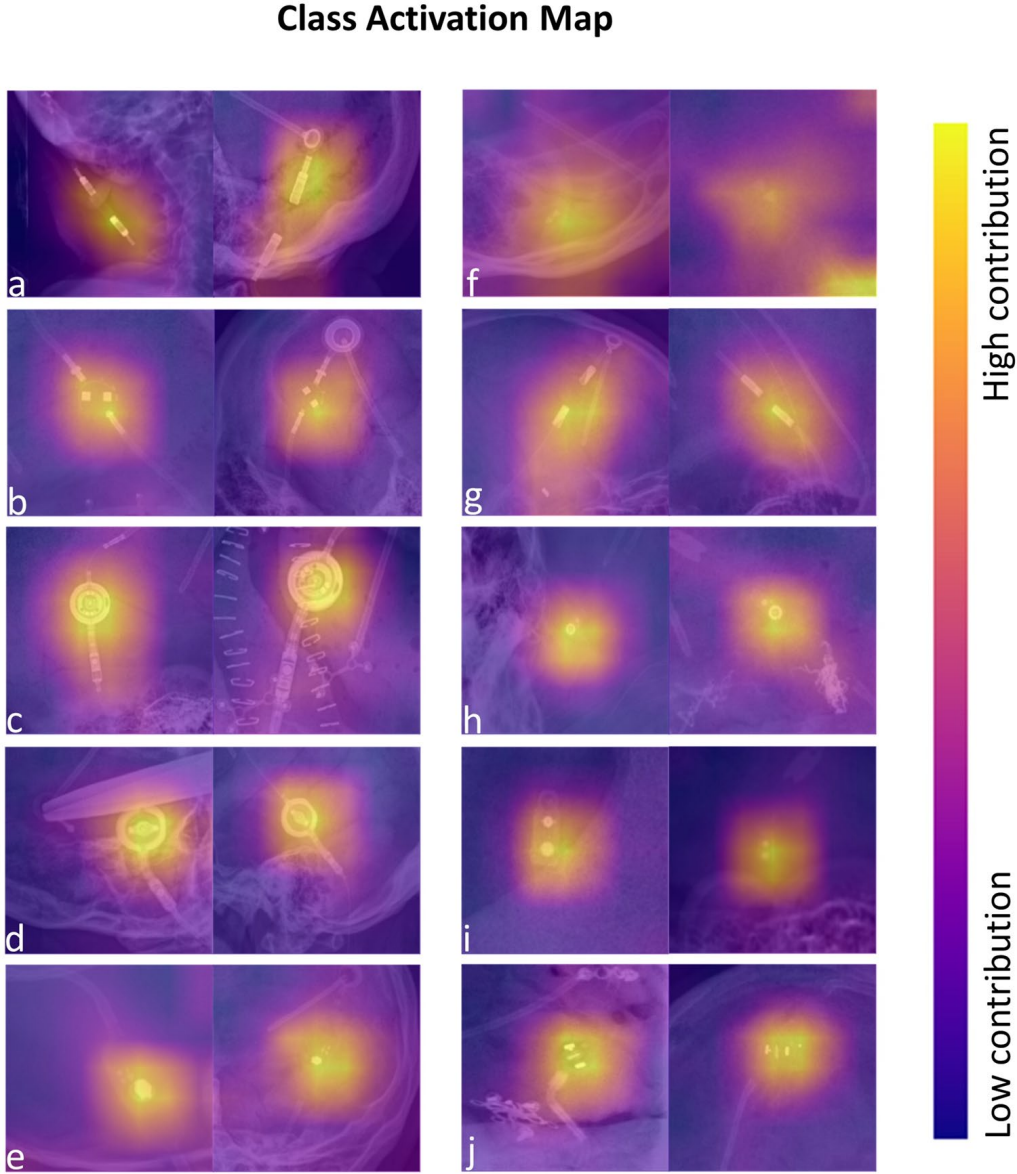
Class activation map (CAM) of two shunt valve models of each shunt valve: (**a**) Integra Spitz-Holter, (**b**) Sophysa Sophy Mini SM8, (**c**) Miethke proGAV 2, (**d**) Miethke proGAV 1, (**e**) Medtronic PS Medical Strata, (**f**) Medtronic PS Medical Delta, (**g**) Integra DP, (**h**) Codman Hakim Programmable, (**i**) Codman Hakim Precision Fixed Pressure, and (**j**) Integra Spitz-Holter

To identify discriminative image regions, we calculated class activation maps (CAM) [21] on two sample images of each shunt valve. Our deep learning model accurately used the location of the shunt valve to make its decision. The predominant shunt valve models in our dataset yielded a more precise CAM in the valve area than the models with a smaller training dataset (Fig. 3).

The least reliable detection was observed with the Sophysa Sophy SM8 valve, which had an F1-score of 92% due to a recall rate of only 86%. Since the dataset contained only 33 Sophysa Sophy SM8 valve images, the weak performance was probably due to the limited amount of training data available for this valve. However, the model did achieve excellent results for some other shunt valves in the lower range, namely the Codman Hakim Precision Fixed Pressure (*n* = 58), Medtronic PS Medical Delta (*n* = 60), Integra Spitz-Holter (*n* = 65), and Integra DP (*n* = 80).

## Discussion

Our model’s validation results demonstrate its ability to accurately distinguish between ten different shunt valve types. In comparison, a previous study using a similar deep learning approach with transfer learning achieved a 96% accuracy in identifying five shunt valve types [6]. Another study reported a detection accuracy of 95% in identifying three shunt valve types using a model trained on smartphone images of X-rays [20]. Therefore, our study presents a more extensive and accurate deep learning model, both in terms of identification accuracy and the range of identifiable shunt valve types, surpassing the results of the previous studies. One major limitation of this study is the limited image dataset, which does not represent all the available shunt valve models. The transfer learning technique used in this study relied on a pre-trained model on ImageNet, which does not include any medical X-ray images. However, our model performed well, indicating the feasibility of using models pre-trained on non-medical datasets for medical imaging tasks. Similar results of using ImageNet pre-trained models on medical X-ray images have been reported previously [5]. This approach seems to be an effective way to train a neural network with only a limited amount of available training images. While our AI-based approach has shown promising results in identifying CSF shunt valves, its implementation in a clinical setting would require a larger dataset for comprehensive validation. Nevertheless, this technology could already be of use to clinicians who are familiar with its limitations and use it judiciously.

With the potential for a larger dataset containing more images, it may be possible to develop an automated system capable of reading CSF shunt valve pressure settings for a given valve. Although our study was limited by the amount of available training data, this presents an exciting opportunity for future research and development.

## Conclusion

Our data indicates that deep learning has the potential to automatically detect different shunt valve models with high accuracy and could facilitate the identification of an unknown shunt valve on X-ray and CT scout images. Such a deep learning model could be directly integrated into a PACS system or standalone application to facilitate clinical workflow. We are currently in the process of extending our dataset and making the deep learning model described here accessible to neurosurgeons and radiologists as an easy-to-use smartphone application to simplify the daily clinical workflow.

## Supplementary Information

Below is the link to the electronic supplementary material.

Supplementary file1 (PDF 339 KB)

Supplementary file2 (PDF 138 KB)

## Data Availability

The dataset is publicly available and can be accessed at https://github.com/CSFShuntvalves/xray_csf_shuntvalves. The source code used for this study is available from the corresponding author upon reasonable request.
