# Supplementary material for: Applied deep learning in neurosurgery: identifying cerebrospinal fluid (CSF) shunt systems in hydrocephalus patients
Source: Acta Neurochir (Wien). 2024 Feb 7;166(1):69. doi: 10.1007/s00701-024-05940-3 (PMC10847194; doi:10.1007/s00701-024-05940-3)
Supplement: Supplementary file 1 — Supplementary file1 (PDF 339 KB) [file 701_2024_5940_MOESM1_ESM.pdf]

Supplementary Material: CLEAR checklist

Section	No.	Item	Yes	No	n/a	Page
Title						
	1	Relevant title, specifying the radiomic methodology	X	☐	☐	1
Abstract						
	2	Structured summary with relevant information	X	☐	☐	1
Keywords						
	3	Relevant keywords for radiomics	X	☐	☐	1
Introduction						
	4	Scientific or clinical background	X	☐	☐	3-4
	5	Rationale for using a radiomic approach	X	☐	☐	4
	6	Study objective(s)	X	☐	☐	4
Method						
Study Design	7	Adherence to guidelines or checklists (e.g., CLEAR checklist)	X	☐	☐	4
	8	Ethical details (e.g., approval, consent, data protection)	X	☐	☐	8
	9	Sample size calculation	☐	☐	X	
	10	Study nature (e.g., retrospective, prospective)	☐	☐	X	
	11	Eligibility criteria	X	☐	☐	4
	12	Flowchart for technical pipeline	X	☐	☐	Figure 1
Data	13	Data source (e.g., private, public)	X	☐	☐	4
	14	Data overlap	X	☐	☐	4
	15	Data split methodology	X	☐	☐	5
	16	Imaging protocol (i.e., image acquisition and processing)	X	☐	☐	4
	17	Definition of non-radiomic predictor variables	☐	☐	X	
	18	Definition of the reference standard (i.e., outcome variable)	X	☐	☐	4
Segmentation	19	Segmentation strategy	☐	☐	X	
	20	Details of operators performing segmentation	☐	☐	X	
Pre-processing	21	Image pre-processing details	X	☐	☐	4
	22	Resampling method and its parameters	☐	☐	X	
	23	Discretization method and its parameters	☐	☐	X	

	24	Image types (e.g., original, filtered, transformed)	X	☐	☐	4
Feature extraction	25	Feature extraction method<br><br>Indicate which software programs or tools are used for radiomic feature extraction. Specify the version of the software and the exact configuration parameters (also see Item#55). Provide reference and web link to the software. Indicate if the software adheres to the benchmarks/certification of IBSI [25]. Specify the general feature types, such as deep features, hand-crafted features, engineered features, or a combination. Refer to the mathematical formulas of the hand-crafted and engineered features. Provide formulas and code if new hand-crafted features are introduced. Present the architectural details for deep feature extraction. Provide details of any feature engineering performed. Specify whether radiomic features are extracted in a two-dimensional (2D) plane, 2D tri-planar, or three-dimensional (3D) space. If 2D features are extracted from 3D segmentation, provide reasons (e.g., large slice thickness) as to why such an approach is followed.	X	☐	☐	4
	26	Feature classes	☐	☐	X	
	27	Number of features	☐	☐	X	
	28	Default configuration statement for remaining parameters	☐	☐	X	
Data preparation	29	Handling of missing data	☐	☐	X	
	30	Details of class imbalance	X	☐	☐	Table 1
	31	Details of segmentation reliability analysis	☐	☐	X	
	32	Feature scaling details (e.g., normalization, standardization)	☐	☐	X	
	33	Dimension reduction details	☐	☐	X	
Modeling	34	Algorithm details	X	☐	☐	5
	35	Training and tuning details	X	☐	☐	5
	36	Handling of confounders	☐	☐	X	
	37	Model selection strategy	☐	☐	X	
Evaluation	38	Testing technique (e.g., internal, external)	☐	☐	X	
	39	Performance metrics and rationale for choosing	X	☐	☐	6
	40	Uncertainty evaluation and measures (e.g., confidence intervals)	☐	☐	X	
	41	Statistical performance comparison (e.g., DeLong's test)	☐	☐	X	
	42	Comparison with non-radiomic and combined methods	☐	☐	X	
	43	Interpretability and explainability methods	☐	☐	X	
Results						
	44	Baseline demographic and clinical characteristics	☐	☐	X	
	45	Flowchart for eligibility criteria	☐	☐	X	
	46	Feature statistics (e.g., reproducibility, feature selection)	☐	☐	X	
	47	Model performance evaluation	X	☐	☐	6

	48	Comparison with non-radiomic and combined approaches	☐	☐	X	
Discussion						
	49	Overview of important findings	X	☐	☐	6-7
	50	Previous works with differences from the current study	X	☐	☐	7
	51	Practical implications	X	☐	☐	7
	52	Strengths and limitations (e.g., bias and generalizability issues)	X	☐	☐	7
Open Science						
Data availability	53	Sharing images along with segmentation data	X	☐	☐	8
	54	Sharing radiomic feature data	☐	☐	X	
Code availability	55	Sharing pre-processing scripts or settings	X	☐	☐	5
	56	Sharing source code for modeling	☐	X	☐	
Model availability	57	Sharing final model files	☐	X	☐	
	58	Sharing a ready-to-use system	☐	X	☐	

Yes, details provided; **No**, details not provided; **n/a**, not applicable
